# Heavy Chronic Intermittent Ethanol Exposure Alters Small Noncoding RNAs in Mouse Sperm and Epididymosomes

**DOI:** 10.3389/fgene.2018.00032

**Published:** 2018-02-08

**Authors:** Gregory R. Rompala, Anais Mounier, Cody M. Wolfe, Qishan Lin, Iliya Lefterov, Gregg E. Homanics

**Affiliations:** ^1^Center for Neuroscience, University of Pittsburgh, Pittsburgh, PA, United States; ^2^Department of Environmental and Occupational Health, University of Pittsburgh, Pittsburgh, PA, United States; ^3^Mass Spectrometry Facility, Center for Functional Genomics, University at Albany, Rensselaer, NY, United States; ^4^Department of Anesthesiology, University of Pittsburgh, Pittsburgh, PA, United States; ^5^Department of Pharmacology and Chemical Biology, University of Pittsburgh, Pittsburgh, PA, United States; ^6^Department of Neurobiology, University of Pittsburgh, Pittsburgh, PA, United States

**Keywords:** ethanol, noncoding RNA, sperm, epididymosomes, epigenetics

## Abstract

While the risks of maternal alcohol abuse during pregnancy are well-established, several preclinical studies suggest that chronic preconception alcohol consumption by either parent may also have significance consequences for offspring health and development. Notably, since isogenic male mice used in these studies are not involved in gestation or rearing of offspring, the cross-generational effects of paternal alcohol exposure suggest a germline-based epigenetic mechanism. Many recent studies have demonstrated that the effects of paternal environmental exposures such as stress or malnutrition can be transmitted to the next generation via alterations to small noncoding RNAs in sperm. Therefore, we used high throughput sequencing to examine the effect of preconception ethanol on small noncoding RNAs in sperm. We found that chronic intermittent ethanol exposure altered several small noncoding RNAs from three of the major small RNA classes in sperm, tRNA-derived small RNA (tDR), mitochondrial small RNA, and microRNA. Six of the ethanol-responsive small noncoding RNAs were evaluated with RT-qPCR on a separate cohort of mice and five of the six were confirmed to be altered by chronic ethanol exposure, supporting the validity of the sequencing results. In addition to altered sperm RNA abundance, chronic ethanol exposure affected post-transcriptional modifications to sperm small noncoding RNAs, increasing two nucleoside modifications previously identified in mitochondrial tRNA. Furthermore, we found that chronic ethanol reduced epididymal expression of a tRNA methyltransferase, *Nsun2*, known to directly regulate tDR biogenesis. Finally, ethanol-responsive sperm tDR are similarly altered in extracellular vesicles of the epididymis (i.e., epididymosomes), supporting the hypothesis that alterations to sperm tDR emerge in the epididymis and that epididymosomes are the primary source of small noncoding RNAs in sperm. These results add chronic ethanol to the growing list of paternal exposures that can affect small noncoding RNA abundance and nucleoside modifications in sperm. As small noncoding RNAs in sperm have been shown to causally induce heritable phenotypes in offspring, additional research is warranted to understand the potential effects of ethanol-responsive sperm small noncoding RNAs on offspring health and development.

## Introduction

Studies examining the cross generational effects of alcohol have primarily focused on maternal alcohol abuse during pregnancy given the severe risk of inducing developmental deficits that typify fetal alcohol syndrome in offspring. Given the long-held belief that fathers only contribute genomic information through the germline, the preconception health of the father has historically been viewed as inconsequential to offspring development. However, a surge of recent preclinical research has triggered a growing interest in how various paternal factors such as stress, diet, and alcohol prior to conception can also affect the offspring phenotype, presumably via epigenetic mechanisms in sperm (Finegersh et al., 2015b; Stuppia et al., 2015; Schagdarsurengin and Steger, 2016).

Various forms of chronic ethanol treatment in male rodents prior to conception have been found to directly affect diverse phenotypes such as body weight, cortical thickness, and even behavioral sensitivity to drugs like amphetamine in the next generation (reviewed in Finegersh et al., 2015b). Recently, we added to this evidence, showing that males exposed intermittently to vapor ethanol over 5 weeks produce male offspring with reduced ethanol drinking behavior, increased ethanol sensitivity and attenuated stress responsivity (Finegersh and Homanics, 2014; Rompala et al., 2016, 2017). Since these studies were performed using isogenic sires that played no role in offspring rearing and development, paternal preconception ethanol may be driving unique changes in offspring behavior through nongenomic mechanisms in sperm. Therefore, greater emphasis should be put on understanding the consequences of paternal alcohol abuse prior to conception and identifying potential epigenetic mechanisms in the germline.

Although sperm DNA is densely packed in the nucleus, sperm are not solely passive carriers of genetic material, but also feature a complex epigenetic machinery. As most histones in sperm are exchanged for protamines during spermatogenesis, and sperm DNA loses most of its methylation at fertilization, identifying sperm-based mechanisms of epigenetic inheritance has been challenging (Heard and Martienssen, 2014). However, in addition to chromatin, sperm have a unique RNA profile enriched with diverse small noncoding RNA species (Ostermeier et al., 2002; Krawetz et al., 2011). These include well-described small RNA classes like microRNA and piRNA as well as under-studied groups like tRNA- and mitochondria-derived small RNAs that are overrepresented in sperm (Peng et al., 2012; Schuster et al., 2016b). As the sperm genome is thought to be transcriptionally quiescent (Kierszenbaum and Tres, 1975), these small noncoding RNAs may instead function during the earliest stages of embryogenesis. Indeed, sperm RNA is delivered to the oocyte (Ostermeier et al., 2004) and recent studies have found that sperm-derived small noncoding RNAs are required for normal embryonic development (Liu et al., 2012; Yuan et al., 2016; Guo et al., 2017).

The earliest evidence for RNA-mediated inheritance demonstrated that a mutation-induced white tail color phenotype in mice could be transmitted to wild type offspring via altered sperm RNA (Rassoulzadegan et al., 2006). Since then, numerous studies have found that sperm small noncoding RNAs are sensitive to various paternal environmental factors including stress, diet and exercise (Rodgers et al., 2013; Gapp et al., 2014; Chen et al., 2016a; de Castro Barbosa et al., 2016; Sharma et al., 2016; Short et al., 2016, 2017). Moreover, in humans, alterations in sperm small noncoding RNAs have been associated with obesity (Donkin et al., 2016) and smoking history (Marczylo et al., 2012). Finally, recent intergenerational studies have shown that cross generational effects of stress and diet can be recapitulated in offspring derived from embryos injected with stress- or diet- altered sperm RNAs, respectively, suggesting a causal role in paternal epigenetic inheritance (Gapp et al., 2014; Grandjean et al., 2015; Rodgers et al., 2015; Chen et al., 2016a).

Ethanol has deleterious effects on several measures of sperm quality in mice such as sperm count, circulating testosterone levels, and overall fertility, and similar effects have been found in alcoholic men (reviewed in La Vignera et al., 2013). Additionally, ethanol has been shown to impact epigenetic mechanisms in sperm. For instance, DNA methylation at imprinting gene loci is reduced in chronic ethanol-treated mice (Knezovich and Ramsay, 2012; Finegersh and Homanics, 2014; Liang et al., 2014) and men with alcohol use disorder (Ouko et al., 2009). However, whether ethanol directly affects small noncoding RNAs in sperm is entirely unknown. This is an important question given the prevalence of alcohol use disorder and the implication of small noncoding RNAs as a causal factor in paternally-linked epigenetic inheritance of complex behavior. Therefore, given the evidence that paternal preconception ethanol exposure has intergenerational effects, we hypothesized that ethanol causes epigenetic reprogramming of sperm small noncoding RNAs.

## Results

### Chronic ethanol exposure shifts the small noncoding RNA profile in sperm

**Figure 1:**
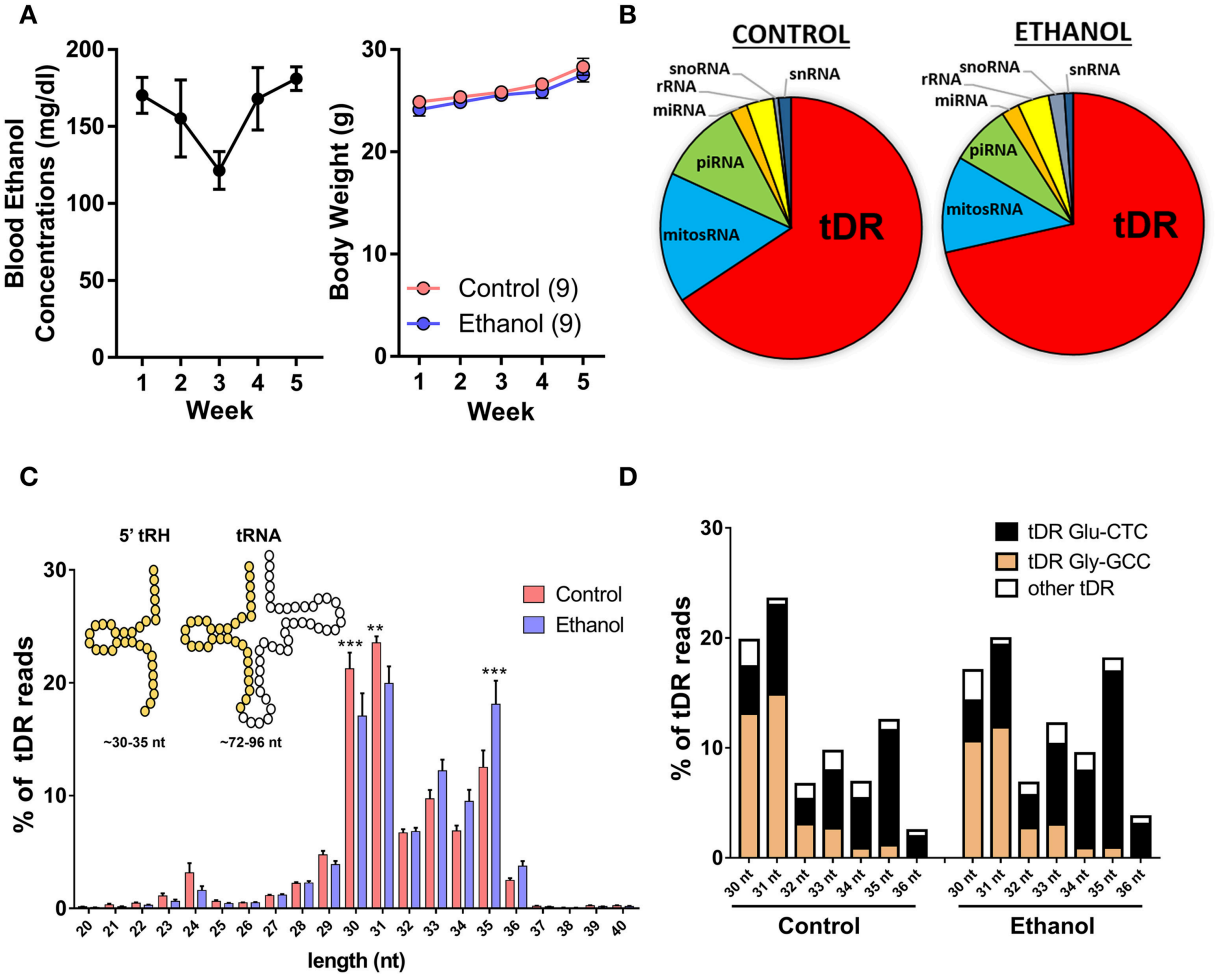
Chronic ethanol shifts the tDR profile of sperm small noncoding RNA. **(A)** Chronic intermittent ethanol vapor exposure (left panel) induced an average blood ethanol concentration of 159.2 ± 9.2 mg/dl [mean (μ) ± standard error of the mean (SEM)] over its 5 week duration. There was no effect of chronic ethanol on body weight (right panel) compared to the control group (*p* > 0.05). (**B)** Pie charts displaying the percentage of each small RNA class represented in sperm from control and ethanol treatment groups. **(C)** Most tDR are 30–35 nt 5′-derived tRNA halves (5′-tRH) (see insert) and chronic ethanol significantly altered the percentage of 30, 31, and 35 nt tDR reads. (**D)** Most 30–36 nt tDR reads map to Glu-CTC and Gly-GCC relative to all other tDR species. Data in bar graphs presented as μ ± SEM. *N* = 9/treatment in all panels. ^**^p < 0.01. ^***^p < 0.001.

Adult male C57BL/6J mice were exposed to vapor ethanol or room air conditions for 8 h/day, 5 days/week over 5 weeks. This chronic ethanol exposure induced an average blood ethanol concentration of ~160 mg/dl and there was no effect of chronic ethanol on body weight at the end of the 5-week exposure (Figure 1A) as previously reported (Finegersh and Homanics, 2014; Rompala et al., 2016). Twenty-four hours following the final ethanol or control exposure, motile sperm were collected from each cauda epididymis for small RNA sequencing. First, we analyzed the various small RNA classes present in mouse sperm. Consistent with other studies in mice (Peng et al., 2012; Sharma et al., 2016), we found the majority (>60%) of 15–45 nucleotide (nt) sequencing reads were transfer RNA (tRNA)-derived small RNAs (tDR) in sperm from both control and ethanol-treated mice while the remaining reads were classified as mitochondrial small RNA (mitosRNA), piRNA, microRNA (miRNA), ribosomal RNA (rRNA), small nucleolar RNA (snoRNA) and small nuclear RNA (snRNA) (Figure 1B). The vast majority of tDR are ~30–35 nt halves (Figure 1C) cleaved from the 5′ end of whole length tRNA at or near the anticodon loop (Supplementary Figure 1). Interestingly, there was a significant interaction between chronic ethanol exposure and the size distribution of tDR reads [*F*_(20, 336)_ = 4.2; *p* < 0.001]. *Post-hoc* analysis revealed that chronic ethanol exposure reduced 30 (*p* < 0.001) and 31 nt tDR (*p* < 0.01) while increasing 35 nt tDR (*p* < 0.001) (Figure 1C). When we sorted tDR by their parent tRNA amino acid and anticodon sequence, two tDR species, Gly-GCC and Glu-CTC, accounted for >70% of all tDR sequencing reads as previously reported (Peng et al., 2012; Chen et al., 2016a; Cropley et al., 2016; Sharma et al., 2016). Notably, the 30–31 nt tDR were dominated by Gly-GCC while Glu-CTC accounted for the majority of 33–35 nt reads (Figure 1D).

### Chronic ethanol exposure alters expression of several small noncoding RNA species

**Figure 2:**
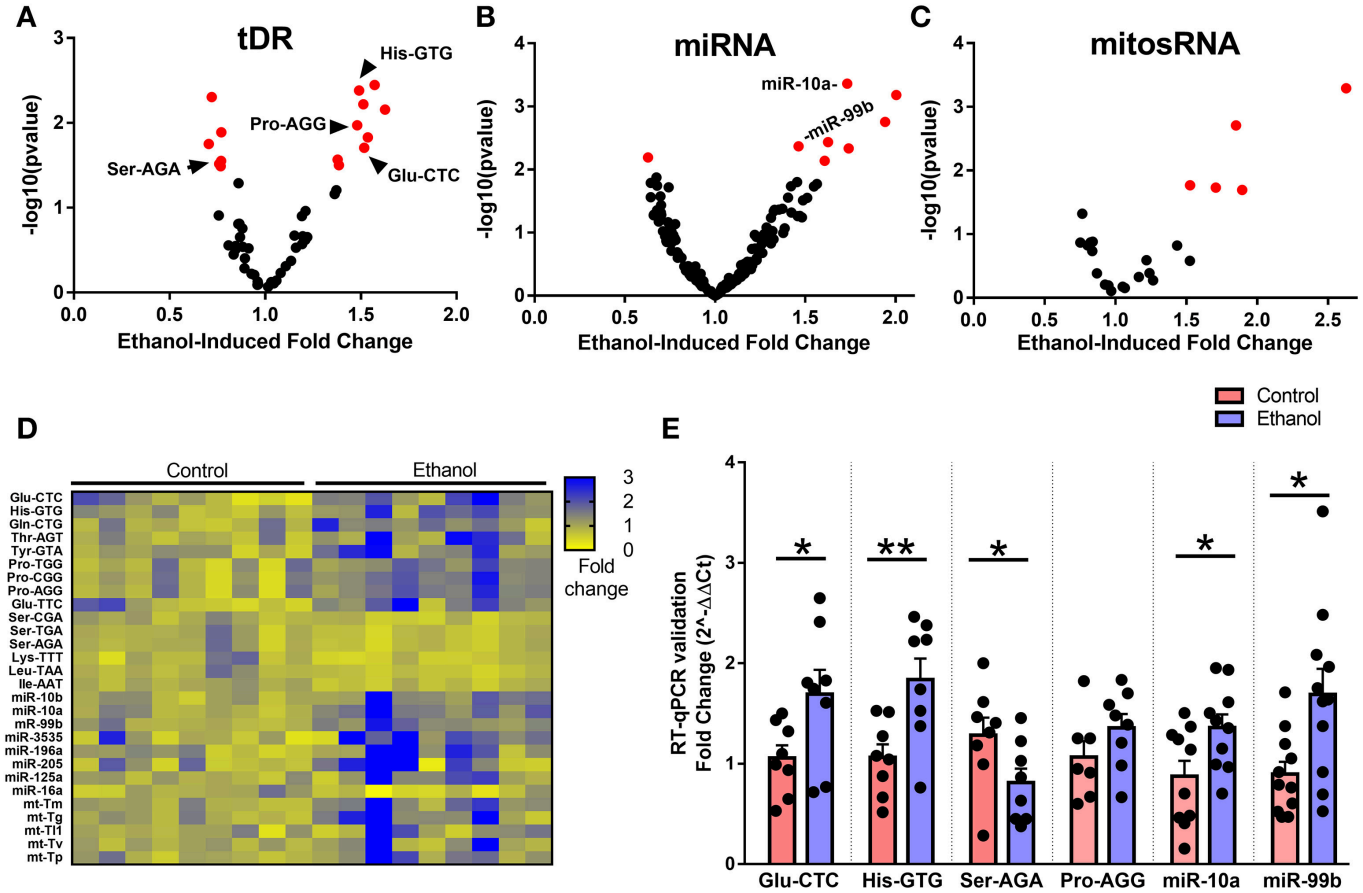
Chronic ethanol alters abundance of several tDR, miRNA, and mitosRNA species in sperm. Volcano plots depicting fold change and log-transformed *p*-value for sperm (**A**) tDR, (**B**) miRNA, and (**C**) mitosRNA. Red dots indicate significance (*q* ≤ 0.1). **(D)** Heat map of differentially expressed sperm small noncoding RNAs representing fold change in normalized counts for each small RNA sequencing sample represented by each column. **(E)** RT-qPCR validation of sequencing results revealed a significant effect of chronic ethanol on sperm tDRs Glu-CTC (*p* < 0.05), His-GTG (*p* < 0.01), Ser-AGA (*p* < 0.05), with no change in Pro-AGG (*p* > 0.05) and significantly increased miR-10a (*p* < 0.05) and miR-99b (*p* < 0.05), *N* = 7–11/treatment. RT-qPCR data presented as μ ± SEM with black dots representing biological replicates (one mouse/replicate). ^*^p < 0.05, ^**^p < 0.01.

When we examined the effect of chronic ethanol exposure on the four major small noncoding RNA types in sperm, small RNA sequencing revealed 15 tDRs (Figure 2A), 8 miRNAs (Figure 2B), 5 mitosRNAs (Figure 2C), and 0 piRNA (Supplementary Figure 2) that were significantly affected by ethanol after false discovery rate adjustment (*q* < 0.1, Figure 2D, Supplementary Table 1). Subsequently, several altered small noncoding RNAs with high endogenous expression were chosen for RT-qPCR validation in an independent cohort of mice. Here, we found that five of the six analyzed small noncoding RNAs were significantly altered by chronic ethanol exposure [increased: tDR Glu-CTC, *t*_(14)_ = 2.33, *p* < 0.05; tDR His-GTG, *t*_(14)_ = 3.14, *p* < 0.01; miR-10a, *t*_(18)_ = 2.41, *p* < 0.05; miR-99b, *t*_(20)_ = 2.79, *p* < 0.05; decreased: tDR Ser-AGA, *t*_(14)_ = 2.08, *p* < 0.05; no change: tDR Pro-AGG, *p* > 0.05] (Figure 2E), supporting the validity of the sequencing results.

### Predicting the functional significance of ethanol-responsive sperm small noncoding RNAs

**Figure 3:**
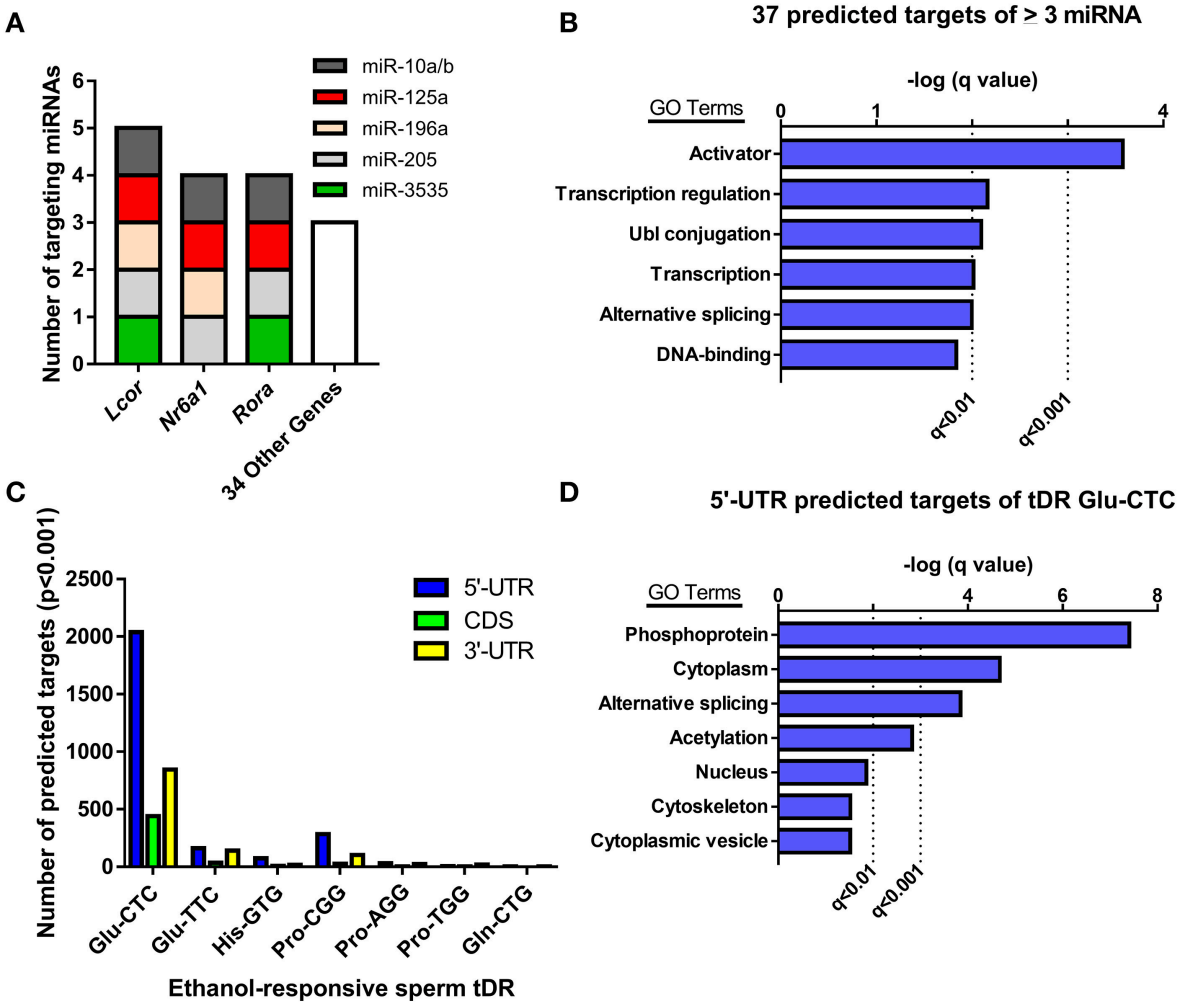
Analyzing predicted gene targets of ethanol-responsive sperm miRNA and tDR Glu-CTC. **(A)** Genes with 3′-UTRs targeted by three or more miRNAs. **(B)** Gene ontology analysis of predicted target genes of ≥ 3 miRNA. **(C)** Number of genes with predicted 5′-UTR, coding, or 3′-UTR targets of ethanol-responsive sperm tDR. (**D**) Gene ontology analysis for genes with predicted 5′-UTR targets of tDR Glu-CTC.

Given the evidence that sperm miRNA and tDR have been causally-linked to paternal epigenetic inheritance, we performed target prediction and gene ontology analysis on these ethanol-responsive small noncoding RNAs to infer functional significance at fertilization. The primary function attributed to miRNAs is RNA silencing through post-transcriptional regulation of the 3′-untranslated region (UTR). Thus, we analyzed the predicted 3′-UTR targets of the 7 miRNA that were increased by chronic ethanol exposure for common targets (Supplementary Table 2). This revealed 37 genes targeted by at least 3 ethanol-enriched miRNAs and 3 genes (*Lcor, Nr6a, Rora*) that were targeted by ≥ 4 (Figure 3A). Gene ontology analysis of the predicted 3′-UTR targets of ≥ 3 sperm miRNA revealed enrichment for activators (i.e., transcription factors), transcription-regulators, and Ubl conjugation genes (*q* < 0.01, Figure 3B, Supplementary Table 3).

Although many studies have found that some tDR species can play a similar role to miRNA in post-transcriptional regulation of gene expression (Keam and Hutvagner, 2015), the specific mechanisms involved are unknown. Recent studies that employed rigorous target prediction analysis for all tDR species suggest that most tDR are more likely to act on the 5′-UTR of transcripts through complementary sequence-based gene regulation (Schuster et al., 2016a,b). Consistently, when we examined the predicted targets of all ethanol-responsive tDR, we found that each tDR examined had a greater number of genes with predicted 5′-UTR targets relative to the coding and 3′-UTR regions (Figure 3C, Supplementary Table 4). Strikingly, the number of genes with predicted 5′-UTR targets was more than 14 times greater for one tDR, Glu-CTC, relative to all other ethanol-responsive tDR examined (Figure 3C, Supplementary Table 4). Given the extreme enrichment of tDR Glu-CTC reads (Figure 1D), in addition to the surfeit of predicted targets, we performed gene ontology on genes with predicted 5′-UTR targets of tDR Glu-CTC, focusing on high confidence results. This revealed enrichment for gene targets associated with signal transduction (i.e., phosphoproteins, acetylation), alternative splicing, and the cytoplasm (*q* < 0.01, Figure 3D, Supplementary Table 5).

### Chronic ethanol exposure alters select sperm small noncoding RNA modifications

**Figure 4:**
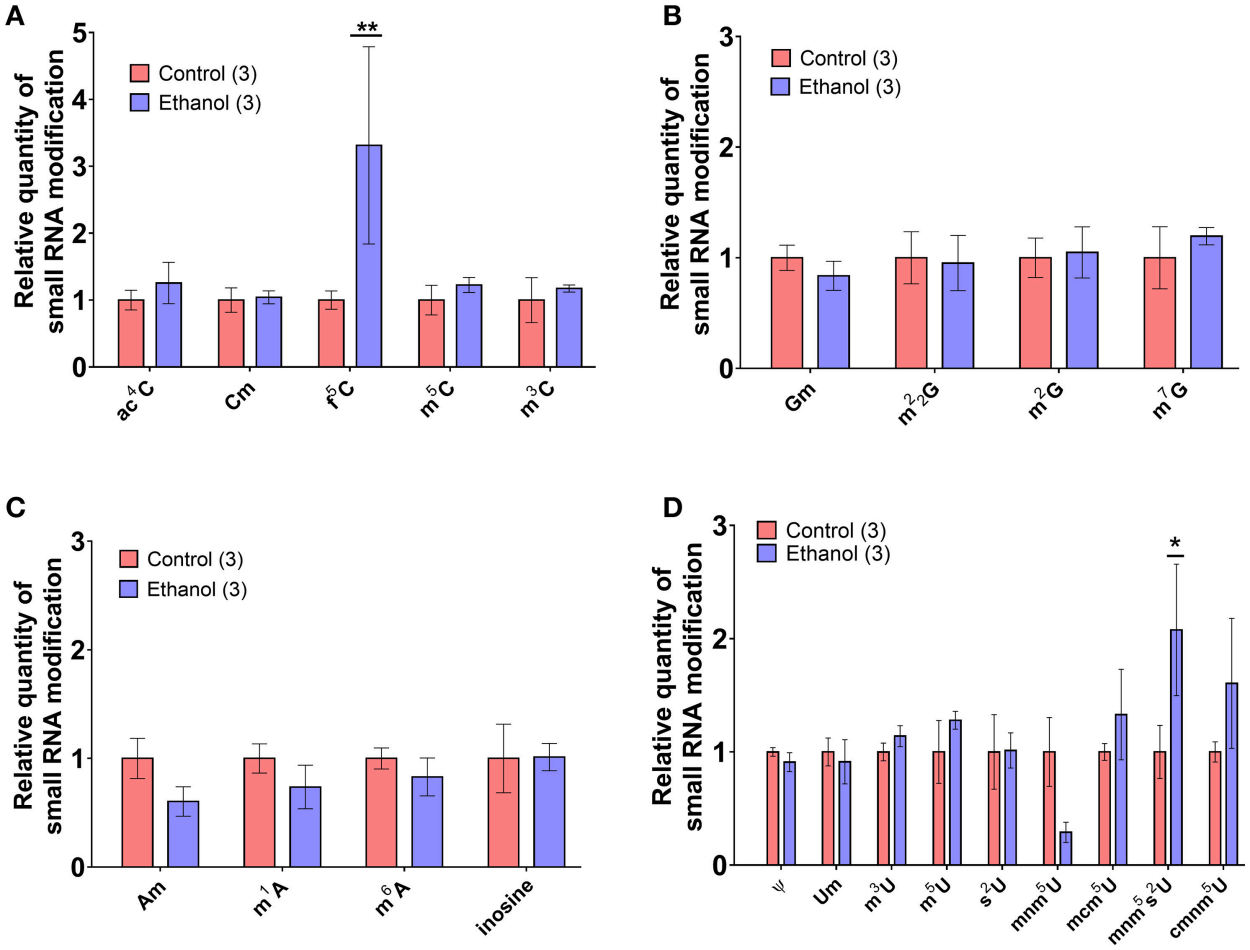
Chronic ethanol alters select RNA modifications in sperm small noncoding RNA. UHPLC-MS/MS was performed on the ~30–40 nt fraction of sperm RNA from chronic ethanol and control exposed groups. Post-transcriptional modifications were examined for each of the parent nucleosides, **(A)** uridine, **(B)** cytidine, **(C)** adenosine, and **(D)** guanosine. Chronic ethanol increased the uridine modification, 5-methylaminomethyl-2-thiouridine (mnm^5^s^2^U) (*q* < 0.1) and the cytidine modification formylcytidine (f^5^C) (*q* < 0.01). Data presented as μ ± SEM bars. *N* = 3 pooled samples/group. ^*^q < 0.1, ^**^q < 0.01.

Recent evidence suggests a functional role for post-transcriptional nucleoside modifications on small noncoding RNAs in sperm, particularly on tDR, as tRNA is the most heavily modified RNA class (Kirchner and Ignatova, 2015). For instance, whereas native sperm tDR is stable in the fertilized oocyte for several hours, synthetic tDR lacking endogenous nucleoside modifications are rapidly degraded (Chen et al., 2016a). Thus, we directly examined if chronic ethanol exposure (see Supplementary Figure 3 for average blood ethanol concentrations and body weights) affects nucleoside modifications in the tDR-enriched ~30–40 nt fraction of sperm RNA using ultra performance liquid chromatography tandem mass spectrometry (UHPLC-MS/MS) (Basanta-Sanchez et al., 2016). We focused our analysis on 22 post-transcriptional modifications previously identified in eukaryotic species (Supplementary Table 6) (Machnicka et al., 2013). This revealed two significantly increased nucleoside modifications: the uridine modification, 5′-methylaminomethyl-2-thiouridine (mnm^5^s^2^U) (*q* < 0.1; Figure 4A) and the cytidine modification, formylcytidine (f^5^C) (*q* < 0.01; Figure 4B). There were no alterations to adenosine (Figure 4C) or guanosine (Figure 4D) base modifications.

### Effects of chronic ethanol on sperm tDR are reflected in epididymosomes

**Figure 5:**
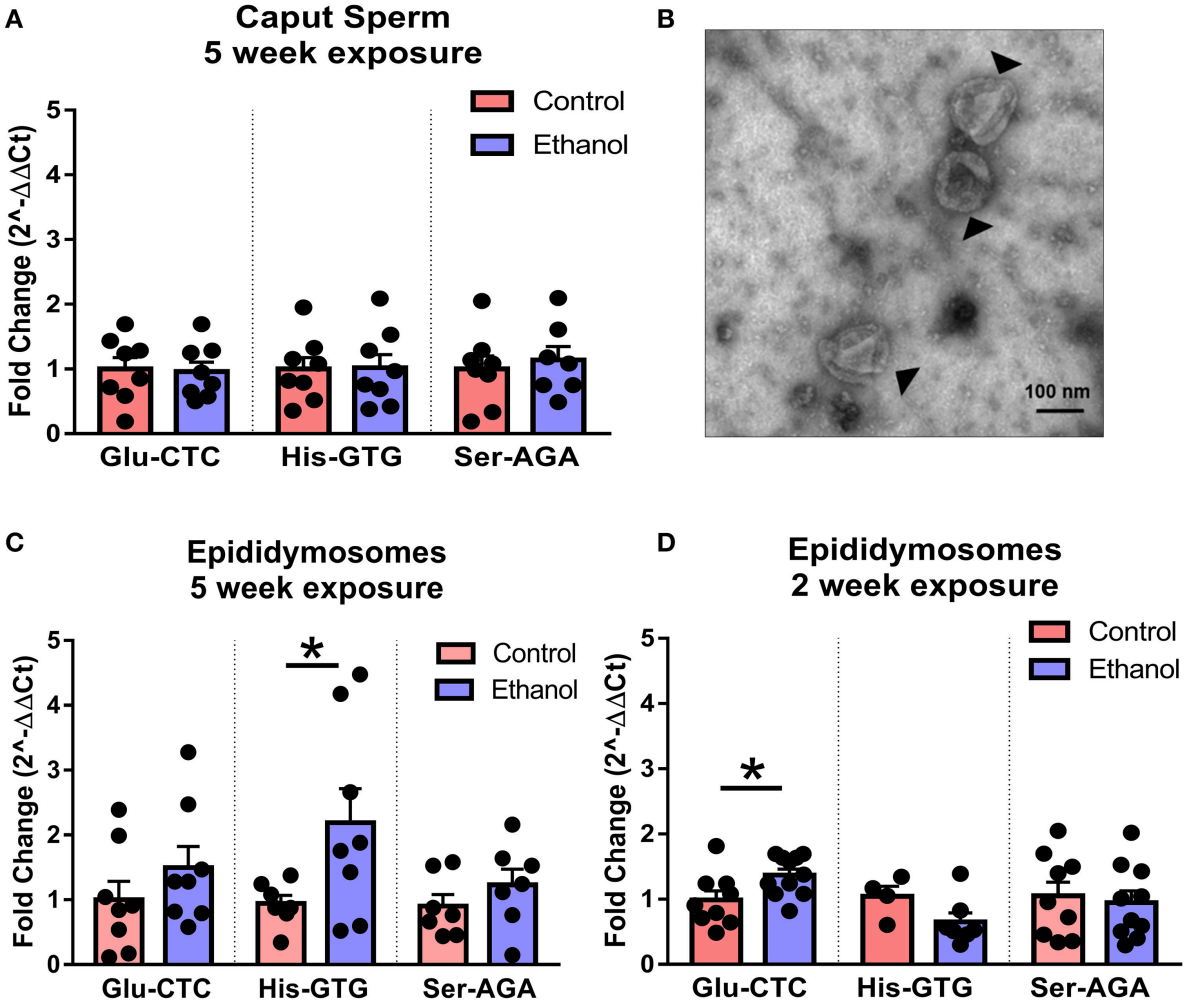
Effects of chronic ethanol on sperm tDR are reflected in epididymosomes. **(A)** RT-qPCR showing no effect of chronic ethanol on tDR Glu-CTC, His-GTG, and Ser-AGA in caput epididymal sperm (*p* > 0.05). (**B**) Transmission electron microscopy image of epididymosomes (arrows) isolated from adult mouse cauda epididymis. **(C)** RT-qPCR reveled a significant effect of chronic ethanol on tDR Glu-CTC with no change in His-GTG or Ser-AGA with 2 weeks of ethanol exposure. **(D)** RT-qPCR showing increased tDR His-GTG with no change in Glu-CTC or Ser-AGA with 5 weeks ethanol exposure. *N* = 4–11/treatment. Data presented as μ ± SEM with black dots representing biological replicates (one mouse/replicate). ^*^p < 0.05.

Following spermatogenesis in the testis, newly developed spermatozoa enter the epididymis, gaining motility while migrating from the caput to cauda segment where mature sperm are stored prior to ejaculation. Interestingly, when we observed sperm isolated from the caput segment, there was no effect of chronic ethanol exposure on the tDR species altered by ethanol in cauda sperm (Figure 5A), suggesting the tDR alterations in mature sperm emerge during epidydimal transit or storage. This is consistent with recent evidence suggesting that sperm tDR are nearly absent in testis and become the dominant small RNA type through interactions with tDR-enriched extracellular vesicles or “epididymosomes” in the epididymal lumen (Reilly et al., 2016; Sharma et al., 2016). Supporting those findings, we found that immature sperm from testis were enriched for tDR species following coincubation with epididymosomes *in vitro* (Supplementary Figures 4A,B). Remarkably, when we isolated cauda epididymosomes from control and ethanol-treated mice (Figure 5B) after 2- and 5-week exposure times and used RT-qPCR to examine the same tDR species that were altered in cauda sperm, tDR Glu-CTC was increased at 2 weeks ethanol treatment [*t*_(18)_ = 2.41, *p* < 0.05; Figure 5C] and tDR His-GTG was increased at 5 weeks [*t*_(13)_ = 2.20, *p* < 0.05; Figure 5D] with no change in tDR Ser-AGA (*p* > 0.05) at either time point. Expression of each tDR was not correlated between cauda sperm and cauda epididymosomes at 5 weeks (Supplementary Figures 5A–C, *p* > 0.05), although there was a significant positive correlation at 2 weeks for tDR Glu-CTC in the ethanol group (Supplementary Figure 5D, *p* < 0.05).

### Chronic ethanol reduces epididymal expression of *Nsun2* gene involved in tDR biogenesis

**Figure 6:**
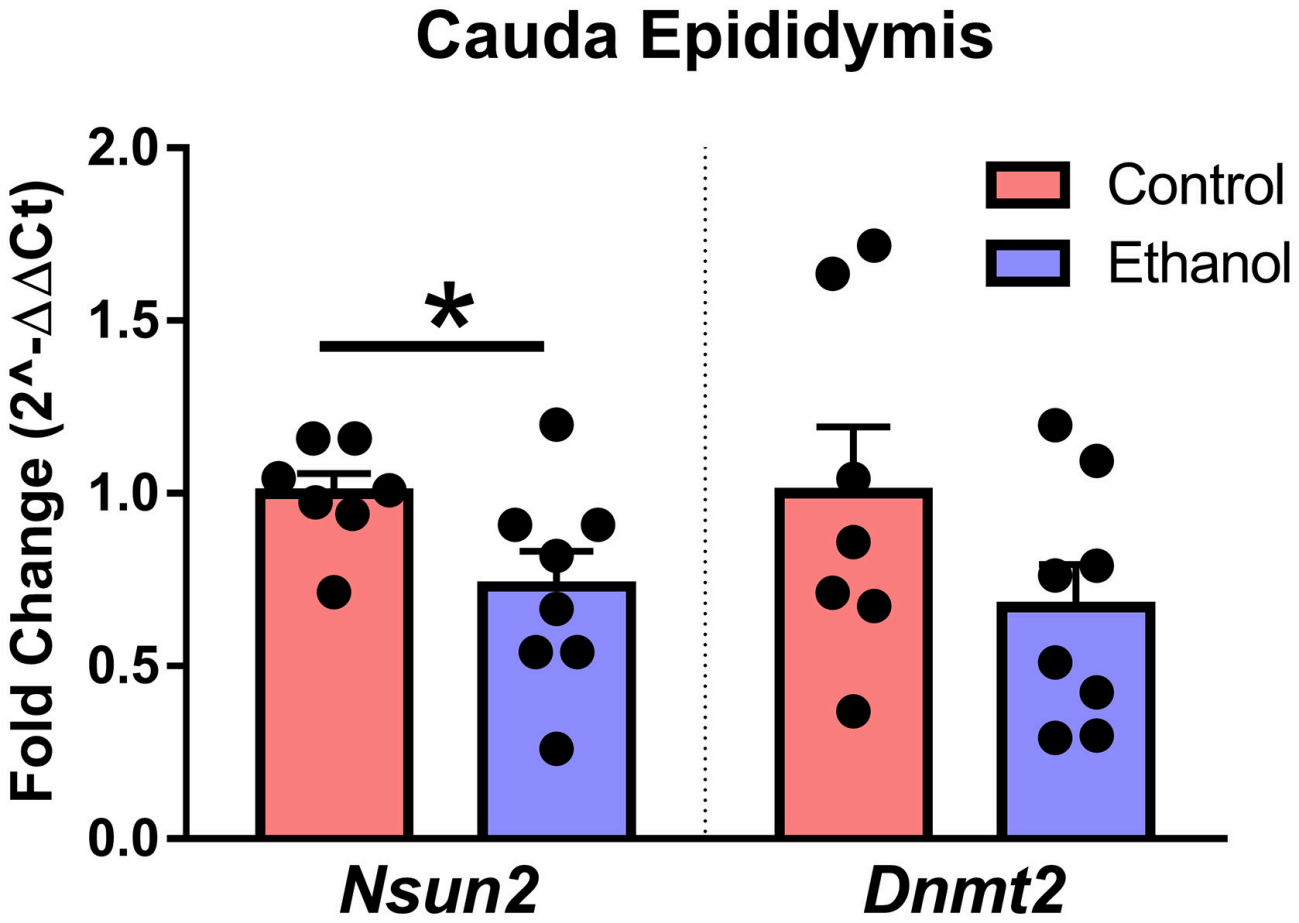
Effect of chronic ethanol on epididymal expression of genes regulating tDR biogenesis. RT-qPCR in cauda epididymal tissue revealed a significant effect of chronic ethanol on the tRNA methyltransferase *Nsun2* (*p* < 0.05) with no change in *Dnmt2* (*p* > 0.5). *N* = 7–8/treatment. Data presented as μ ± SEM with black dots representing biological replicates (one mouse/replicate). ^*^p < 0.05.

Although the specific mechanisms in sperm and epididymis are unknown, loss of the tRNA cytosine-5 methyltransferases *Nsun2* or *Dnmt2* increases angiogenin-dependent cleavage of tRNA into 5′-derived tRNA halves (Schaefer et al., 2010; Blanco et al., 2014), the primary tDR subtype in sperm. Interestingly, we found that chronic ethanol exposure reduced expression of *Nsun2* [*t*_(13)_ = 2.2, *p* < 0.05] with no effect on *Dnmt2* (*p* > 0.05) in cauda epididymis (Figure 6).

## Discussion

Several studies suggest that small noncoding RNAs are functional epigenetic regulators in sperm, capable of directing gene expression in the early embryo and ultimately impacting offspring behavior into adulthood. The current study is the first to our knowledge to examine the effects of ethanol on small noncoding RNAs in sperm. We found that chronic intermittent ethanol exposure altered the expression of several sperm tDR, mitosRNAs, and miRNAs. In addition, chronic ethanol increased specific posttranscriptional nucleoside modifications on sperm small noncoding RNAs. Gene ontology analysis of predicted ethanol-responsive miRNA and tDR targets revealed enrichment for gene sets involved in diverse biological functions, most robustly transcriptional factors and phosphoproteins. Finally, in the epididymis, we found that ethanol-responsive sperm tDR were similarly affected in extracellular vesicles (i.e., epididymosomes) and chronic ethanol exposure reduced expression of a tRNA methyltransferase, *Nsun2*, directly involved in tDR biogenesis (Blanco et al., 2014), suggesting a somatic origin to altered small noncoding RNAs in the male germline.

Many psychiatric and addiction disorders including alcohol use disorder have long been faced with what's been termed the “missing heritability” problem. That is, while alcohol use disorder has ~50% heritability (Prescott and Kendler, 1999; Young-Wolff et al., 2011; Ystrom et al., 2011), putative genetic variants associated with alcoholism account for only a minor fraction of that heritability (Treutlein and Rietschel, 2011). This suggests a significant role for non-genomic germline mechanisms of inheritance. The finding that chronic ethanol exposure alters sperm small noncoding RNAs adds to a growing literature demonstrating that a diverse range of paternal preconception exposures with cross-generational effects are associated with altered small noncoding RNAs in sperm (Rodgers et al., 2013; Gapp et al., 2014; Chen et al., 2016a; de Castro Barbosa et al., 2016; Sharma et al., 2016; Short et al., 2016, 2017). Remarkably, recent studies identified a causal relationship between altered small noncoding RNA and intergenerational phenotypes (Gapp et al., 2014; Grandjean et al., 2015; Rodgers et al., 2015; Chen et al., 2016a). We have shown previously that chronic preconception ethanol exposure alters complex behaviors in male offspring including ethanol drinking preference and stress responsivity (Finegersh and Homanics, 2014; Rompala et al., 2016, 2017). Here we found the same chronic ethanol exposure induces differential expression of several small noncoding RNA species in sperm. Thus, additional studies are needed to directly test the role of ethanol-responsive sperm small noncoding RNAs in the heritable effects of paternal preconception chronic ethanol exposure.

Among the different small noncoding RNA types, we found that tDR were most affected by chronic ethanol treatment. Given the abundance of tDR in sperm and emerging evidence of their role in gene expression regulation, tDR have become a major focus as a potential causal mechanism for paternally-linked epigenetic inheritance. Low protein diet, high fat diet, and increased exercise have all been shown to directly confer changes in sperm tDR (Chen et al., 2016a; Sharma et al., 2016; Short et al., 2017) while obesity and vinclozolin exposure affect sperm tDRs transgenerationally (Cropley et al., 2016; Schuster et al., 2016a). Remarkably, one recent study found that the effects of paternal high fat diet on glucose tolerance in offspring were recapitulated in mice derived from fertilized embryos injected with sperm tDR, but not when the embryos were injected with all other sperm RNA classes except tDR (Chen et al., 2016a). This illustrates a specific causal role for tDR in RNA-mediated epigenetic inheritance.

In addition to affecting tDR, chronic ethanol exposure increased several of the second most abundant small noncoding RNA type, mitosRNA. While little is known about these small noncoding RNAs, they are derived from mitochondrial genes for rRNA and tRNA and have been shown to increase the expression of their parent genes *in vitro* (Ro et al., 2013). Given that mitosRNA are enriched in total sperm and yet barely detected in the sperm head (Schuster et al., 2016b), they are likely confined to the sperm mitochondrial sheath. Several parameters of mitochondrial function appear critical for sperm motility and fertilization capacity, including control of reactive oxygen species production, apoptotic pathways, and calcium homeostasis (Amaral et al., 2013). As chronic ethanol has been shown to affect several measures of sperm quality including reduced motility and increased apoptosis (Rahimipour et al., 2013), it is possible that ethanol directly affects sperm mitochondrial function. While paternal mitochondria do enter the oocyte at fertilization, the mitochondria and its DNA are rapidly degraded (Politi et al., 2014). It is unknown whether the mitochondrial RNA and mitosRNAs are similarly degraded or if they may serve some function in the early embryo.

While less expressed in sperm relative to other small noncoding RNA species, miRNA have been found to play a critical role in fertilization and preimplantation development (Liu et al., 2012; Yuan et al., 2016). Furthermore, altered sperm miRNA have been associated with the greatest range of environmental factors (although this may be due to the greater use of miRNA-specific analysis strategies such as microarray). Most notable among these studies, chronic variable stress was shown to increase nine sperm miRNAs and offspring of stressed sires have a blunted stress responsivity phenotype (Rodgers et al., 2013). That same phenotype could be elicited in mice derived from control fertilized embryos injected with the nine stress-enriched miRNAs, suggesting a causal role for environmentally-responsive sperm miRNA (Rodgers et al., 2015). Interestingly, although we previously found that our chronic ethanol exposure similarly blunted stress responsivity in male offspring (Rompala et al., 2016), none of the stress-enriched miRNAs were affected in the current study, suggesting the intergenerational effects of the current chronic ethanol exposure are likely conferred through a different constellation of small noncoding RNAs or an alternative epigenetic pathway.

Although the specific function of sperm small noncoding RNAs is unknown, the two species directly implicated in intergenerational inheritance, miRNA and tDR, are both associated with post-transcriptional regulation of gene expression and are hypothesized to exert their epigenetic effects by regulating transcriptional cascades in the fertilized oocyte (Chen et al., 2016b). As miRNA have been found to predominantly target the 3′-UTR of mRNAs, we used sequence homology target prediction to identify common 3′-UTRs targeted by ethanol-responsive sperm miRNAs. Gene ontology analysis of common targets revealed enrichment for transcription factors and transcriptional regulators. Three genes, *Lcor, Nr6a1*, and *Rora*, were targeted by four or more of the seven ethanol-enriched miRNAs. *Lcor* binds with various steroid receptors including estrogen, progesterone, and glucocorticoid receptors (Palijan et al., 2009). It has been found to directly attenuate progesterone regulated gene expression (Fernandes et al., 2003) and is highly expressed in two-cell embryos (Fernandes et al., 2003), suggesting a critical role in steroid-hormone receptor mediated gene expression during embryogenesis. In addition, loss of *Nr6a1*, also known as germ cell nuclear factor, results in lethality during embryonic development (Wang and Cooney, 2013). Thus, future studies are warranted to investigate the effects of paternal chronic ethanol exposure on predicted targets of ethanol-responsive miRNA during embryogenesis.

While the specific role of sperm tDR is unknown, most evidence suggests tDR act similarly to miRNA via post-transcriptional regulation of mRNA. Notably, while miRNA function is primarily associated with regulation at 3′-UTRs, tDR are more likely to target 5′-UTRs (Schuster et al., 2016a,b). Remarkably, we found that the number of predicted 5′-UTR targets was substantially greater for one tDR, Glu-CTC, relative to all other analyzed species. This was striking considering it is also enriched several hundred-fold relative to nearly all other small noncoding RNAs in sperm. Gene ontology analysis of predicted 5′-UTR targets of tDR Glu-CTC revealed greatest enrichment for genes related to phosphoprotein, alternative splicing, cytoplasm, and acetylation. Therefore, Glu-CTC is well-positioned to be functionally significant in the fertilized oocyte. Supporting this notion, one study found that expression of approximately half of the predicted mRNA targets of tDR Glu-CTC was reduced more than two-fold from the oocyte to four cell-stage of embryonic development (Cropley et al., 2016). Furthermore, injecting the other equally-enriched tDR species in sperm, Gly-GCC, into fertilized oocytes dramatically altered gene expression while an equal amount of endogenously less-expressed sperm tDRs were comparatively ineffective (Sharma et al., 2016), suggesting a potentially greater role for sperm small RNAs with robust endogenous expression such as Glu-CTC. Future studies will need to examine the effect of ethanol-sensitive tDR Glu-CTC on gene expression in the early embryo.

There is growing interest in the role of post-transcriptional nucleoside modifications in RNA function. Small noncoding RNAs also feature these modifications which are important for stability in the oocyte and even the ability of small noncoding RNAs to induce intergenerational phenotypes (Chen et al., 2016a). When we used HPLC-MS/MS to examine the tDR enriched ~30–40 nt sperm RNA fraction directly for nucleoside modifications, we found a significant effect of chronic ethanol exposure on two modifications, f^5^C and mnm^5^s^2^U. Each of these nucleoside modifications have been identified previously on intact mitochondrial-encoded tRNAs at the wobble position of the anticodon loop (Yan and Guan, 2004; Machnicka et al., 2013; Nakano et al., 2016), critical to tRNA structure and codon recognition. Two pathogenic point mutations have been associated with the inability to form f^5^C modifications (Nakano et al., 2016), suggesting functional significance. Whether these nuceloside modifications reflect alterations to the parent mitochondrial tRNA in sperm or if they also serve a specific function on mitosRNA such as stability or target recognition is unknown. Interestingly, f^5^C is found on mt-Tm, the mitochondrial tRNA for methionine (Nakano et al., 2016), and mitosRNAs mapping to mt-Tm were increased by chronic ethanol exposure (Figure 2D). Increased f^5^C may be a consequence of increased mt-Tm small noncoding RNAs or it is also possible that f^5^C stabilizes mt-Tm-mapping mitosRNAs. Overall, these findings further support the notion that in addition to sperm small RNA abundance, post-transcriptional modifications are sensitive to environmental insults such as chronic ethanol exposure.

Given that DNA in sperm is condensed by highly alkaline protamines, there is minimal transcriptional activity in mature sperm. Thus, environmentally-induced changes to the sperm RNA profile are likely driven by extracellular factors. Supporting this notion, the epididymis is enriched with principal secretory cells that release extracellular vesicles (i.e., epididymosomes) capable of fusing with the sperm membrane. Many studies have characterized epididymosome-mediated protein exchange with sperm (reviewed in Sullivan, 2016). More recently, it was found that epididymosomes carry a tDR-enriched small RNA milieu that is similar to sperm (Sharma et al., 2016) and epididymosomes can directly transfer small noncoding RNAs to immature sperm *in vitro* (Reilly et al., 2016; Sharma et al., 2016). Here, we found that caput sperm did not have the same ethanol-induced changes to sperm tDR seen in cauda sperm, suggesting ethanol-sensitive sperm tDRs are altered during epididymal transit. Indeed, when we examined RNA from epididymosomes, we found that the effects of chronic ethanol exposure on sperm tDRs Glu-CTC and His-GTG were reflected in epididymosomes. While we only found a correlation between tDR and epididymosomes for tDR Glu-CTC and only following two, but not 5 weeks of ethanol exposure, several factors may contribute to differences between the RNA cargo of epididymosomes vs. sperm stored in the cauda epididymis at a given time point. For instance, after epididymal transit, mature rodent sperm are estimated to remain motile in the cauda epididymis for 1 month (Jones, 1999). Moreover, the majority of epididymosomes have been found to target dead sperm while a subtype of CD9-positive epididymosomes show increased preference for live sperm (Caballero et al., 2013). Thus, a better understanding of the temporal and subtype-specific dynamics of *in vivo* epididymosome to sperm RNA transfer is needed in future investigations of this novel soma to germline mechanism.

In other tissues, the production of 5′-derived tRNA halves results from cellular stress-induced cleavage at the anticodon loop by the RNase angiogenin (Fu et al., 2009). This tRNA cleavage is increased in the absence of cytosine-5 methylation (Tuorto et al., 2012). The major cytosine-5 tRNA methyltransferase enzymes are *Nsun2* and *Dnmt2* and chronic ethanol exposure reduced expression of *Nsun2* in cauda epididymis. Loss of *Nsun2*-dependent tRNA methylation results in dramatically increased cleavage of tRNAs into ~30–35 nt halves by angiogenin (Blanco et al., 2014). Although it is unclear whether angiogenin-mediated cleavage and tRNA cytosine-5 methylation similarly regulate tDR production in epididymis and sperm, *Nsun2* and *Dnmt2* are highly expressed in both testis and epididymis and *Nsun2* is critical for proper germ cell differentiation (Hussain et al., 2013). Furthermore, a recent study found that maternal and paternal *Dnmt2* expression is essential in two separate animal models of RNA-mediated inheritance (Kiani et al., 2013). Thus, tRNA cytosine-5 methyltransferase activity may be important for sperm tDR biogenesis and RNA-mediated epigenetic inheritance. More studies are needed to investigate the mechanistic role of tRNA cytosine-5 methyltransferase enzymes specifically in sperm tDR production and function.

While the emerging evidence suggesting a causal role for small noncoding RNA in inheritance of paternal preconception environment is intriguing, it is important to acknowledge the limitations and remaining challenges to discerning functional significance. For instance, the sperm RNA payload is minuscule (100 fg in rodents) and even the contribution of sperm-enriched tDR and miRNA is negligible relative to the amount of pre-existing copies in the oocyte (Yang et al., 2016). Therefore, studies to date examining the function of specific RNAs in fertilized embryos may not reflect physiological conditions. It is possible that sperm RNAs acquire unique functionality by forming as-yet unidentified protein-RNA effector complexes or through RNA modifications (Kiani et al., 2013). In addition, if the RNA cargo from epididymosomes adhering to the sperm exterior is also trafficked to the embryo cytoplasm, this would greatly increase the paternal RNA contribution (Sharma and Rando, 2017). Finally, sperm-derived RNAs may be reverse-transcribed and amplified in the early embryo as transcriptionally-competent cDNA (Spadafora, 2017). Undoubtedly, additional studies are needed to uncover potential mechanisms of RNA-mediated inheritance.

In summary, our findings provide the first evidence that chronic ethanol exposure alters small noncoding RNA abundance and nucleoside modifications in sperm. Additionally, we provide evidence that ethanol directly alters the same small noncoding RNAs in epididymosomes, further supporting the hypothesis that alterations to sperm RNA may be downstream of environmentally-induced changes to extracellular vesicles in the epididymal lumen. Given the prevalence of alcohol use disorder in men, these findings have significant public health implications. Future studies are needed to directly interrogate the effects of ethanol-sensitive small noncoding RNAs in sperm on embryo and offspring development.

## Methods

All experiments were approved by the Institutional Animal Care and Use Committee of the University of Pittsburgh and conducted in accordance with the National Institutes of Health Guidelines for the Care and Use of Laboratory Animals. Seven-week-old, ethanol-naïve, specific pathogen free C57BL/6J mice were purchased from the Jackson Laboratory (Bar Harbor, ME). Mice were habituated to the University of Pittsburgh animal facility for at least 1 week prior to initiation of experiments. Mice were housed under 12 h light/dark cycles and had *ad libitum* access to food (irradiated 5P76 ProLab IsoPro RMH 3000, [LabDiet, St.Louis, MO]) and water.

### Chronic intermittent ethanol inhalation

Chronic intermittent ethanol inhalation was performed as previously described (Finegersh and Homanics, 2014; Finegersh et al., 2015a; Rompala et al., 2016, 2017). Briefly, 8-week-old male C57BL/6J mice were randomly assigned to one of two treatments. Half the mice were treated in ethanol inhalation chambers in the home cage with water and food for 5 weeks from 09:00 to 17:00 over five consecutive day blocks with 2 days in between blocks. The other half of mice were assigned controls that were exposed to identical chamber conditions without ethanol vapor. Animals were group-housed throughout the exposure and cages, food, and water were all changed routinely after the final exposure of each week. Blood ethanol concentration was measured after the final exposure of each week by extracting tail vein blood using heparin-coated capillary tubes (Drummond, Broomall, PA) and running plasma samples (extracted from blood by centrifugation at 2,300 × g for 10 min) on an Analox EtOH analyzer (AM1, Analox Instruments, London, UK). Tail blood was drawn from all groups to control for the extraction procedure. Ethanol content in the ethanol inhalation chambers was monitored using a custom sensor generously provided by Brian McCool and flow rates in the chambers were adjusted weekly based on blood ethanol concentration measurements made during the preceding week. Importantly, animals do not lose significant body weight (defined as >10%). In addition, the effects of ethanol vapor on lungs, heart, and liver are comparable to those associated with other chronic ethanol exposure models (Mouton et al., 2016).

### Epididymis collection and sperm isolation

Sperm samples were isolated from adult male mice sacrificed ~16–19 h following the final ethanol or room air exposure during the light cycle (08:00–11:00). Briefly, after euthanasia by CO2 asphyxiation, left and right cauda epididymides were dissected into 1.5 ml of EmbryoMax Human Tubal Fluid (HTF) (Sigma-Aldrich, St. Louis, MO) at 37°C. Several small cuts were made in each epididymis to release the sperm into solution. The sperm solution was then transferred to a 1.5 ml Eppendorf tube and motile sperm dispersed in media for 20 min at 37°C. The top 1.2 ml supernatant was carefully collected for further processing while the settled epididymal tissue was stored at −80°C for later RNA extraction. Next, the supernatant was centrifuged at 2,000 × g for 5 min to pellet the sperm. The supernatant from this step was saved for epididymosome isolation and the pelleted sperm was then gently resuspended by pipetting in 1.0 ml of somatic cell lysis buffer (0.1% SDS, 0.5% Triton-X) which was put on ice for 20 min. This step is also critical for lysis and removal of adherent RNA-containing extracellular vesicles (Sharma et al., 2016). Next, the sperm was re-pelleted and washed twice with ice cold 1X PBS. After the final wash, the sperm pellet was lysed in 1.0 ml Trizol (Thermo Fisher, Waltham, MA) supplemented with 200 mM β-mercaptoethanol (Sigma-Aldrich) to facilitate lysis of disulfide-bond enriched sperm cells. Samples were lysed using a 2.0 ml Dounce glass tissue homogenizer to break up the sperm pellet and further homogenized with a mechanical homogenizer on ice followed by brief heating at 65°C for 5 min before being moved back to ice. Complete lysis of the sperm nucleus was confirmed with light microscopy.

Caput sperm were extracted from caput epididymis into 1.5 ml HTF at 37°C. Since caput sperm are not fully motile, sperm were centrifuged at 300 × g for 3 min to discard larger tissue pieces (while the partially motile sperm remained in suspension) and treated with somatic cell lysis buffer for 30 min to enrich for caput sperm and remove adherent epididymosomes. Sperm were then re-centrifuged at 2,000 × g for 5 min and washed twice with 1X PBS. Sample purity was confirmed using light microscopy.

### RNA extraction

All samples were lysed in Trizol (note the additional steps used for sperm described above) using phenol-chloroform separation. The aqueous phase was then processed with Zymo RNA Clean and Concentrator Kit with DNAse1 on-column treatment (Zymo Research, Irving, CA). Final sperm RNA concentrations were determined with Qubit RNA HS assay (Thermo Fisher) and RNA Analysis ScreenTape (Agilent, Santa Clara, CA) was used to confirm absence of 18S and 28S ribosomal peaks that are indicative of somatic cell contamination.

### Small RNA sequencing

Barcoded small RNA libraries were prepared from 100 ng total RNA from individual mice using NEBNext Small RNA for Illumina Kit (New England Biolabs, Ipswich, MA) per manufacturer's instructions. Samples were selected for sequencing (*N* = 9/treatment) by randomly selecting 3 mice from each of 3 cages of 4 mice per treatment group. To prevent carry over of adapter dimers and nonspecific amplicons into the sequencing run, cDNA libraries were size-selected using 2% agarose gel electrophoresis with a Pippin Prep system (Sage Science, Beverley, MA). cDNA libraries were multiplexed and sequenced to an average depth of 9 million reads/sample on a NextSeq500 (Illumina, San Diego, CA) at the John G. Rangos Sr. Research Center at Children's Hospital of Pittsburgh of UPMC (Pittsburgh, PA). Investigators were blinded to treatment for both library preparation and sequencing.

### Sequencing analysis and bioinformatics

Small RNA sequencing fastq files were filtered for read quality and trimmed with Cutadapt (Martin, 2011) which removed library preparation adapters and sequences outside the 15–45 nt range. For alignment to the mouse genome (GRCm38/mm10 assembly), Bowtie2 (Langmead and Salzberg, 2012) was used with standard parameters (–n 1, –l 18, –e 70). Mapped reads were annotated to small noncoding RNA features provided at spermbase.org (Schuster et al., 2016b) and summated with FeatureCounts (Liao et al., 2014). Final normalized counts were extracted and analyzed for differential expression analysis using DESeq2 (Love et al., 2014). For tDR analysis, all sized fragments mapping to a single species (e.g., tDR Glu-CTC) were summed to a single data point. The program tDRmapper (Selitsky and Sethupathy, 2015) was used to determine the size distribution of tDR reads and to further classify tDR species by type of fragmentation (e.g., 5′-tRH).

To predict genes with 3′-UTR targeted by miRNAs, we used TargetScan Mouse Custom ver. 5.2 (Lewis et al., 2005). For an unbiased prediction of genes with 5′UTR, coding or 3′UTR regions targeted by tDR, we used RNAhybrid (Kruger and Rehmsmeier, 2006) with established parameters (Schuster et al., 2016a). Gene ontology analysis was performed on all predicted target gene lists using DAVID Bioinformatics Resources ver. 6.8 (Huang da et al., 2009).

### Reverse-transcription quantitative PCR (RT-qPCR)

For cDNA preparation of tDRs and mRNAs, cDNA was produced using total RNA from individual mice with RevertAid First Strand cDNA Synthesis Kit (Thermo Fisher, Waltham, MA) with gene-specific RT primers (see: Kramer, 2011 for stem-loop primer design methods) for tDR and oligo-dT RT primers for mRNA. For miRNA, cDNA was produced using miScript II RT Kit (Qiagen, Valencia, CA). Diluted cDNA was used for qPCR with iScript SYBR green (BioRad, Hercules, CA) on a BioRad iCycler. Expression was calculated using the 2^−ΔΔCt^ method. Small RNAs and mRNAs were normalized to U6 and β-Actin, respectively. All qPCR amplicons were validated by melt curve analysis, electrophoresis, and, for tDRs, additionally with Sanger sequencing. See Supplementary Materials for a full list of RT-qPCR oligos (Supplementary Table 7).

### Ultra-high-performance liquid chromatography tandem mass spectrometry (UHPLC-MS/MS) analysis of sperm small noncoding RNA modifications

Sperm total RNA was pooled from 4 to 8 mice (3 pooled samples/group), loaded (~1 μg/lane) on Novex TBE-Urea 15% polyacrylamide gels (Thermo Fisher) and electrophoresed at 180 V for 1 h. Under UV light, the ~30–40 nt band of RNA was recovered using ZR small-RNA PAGE Recovery Kit (Zymo Research). For each sample, 100 ng of the recovered small RNA was digested and prepared for UHPLC-MS/MS at the University at Albany RNA Mass Spectrometry Core (Albany, NY) using established methods (Basanta-Sanchez et al., 2016). Briefly, prior to UHPLC-MS/MS analysis, each sample was diluted to 10 ng/μl in 10 μl volume prior to enzymatic hydrolysis. This process involved the use of two enzymes. Nuclease P1 at 37°C overnight first followed by the addition of bacterial alkaline phosphatase at 37°C for 2-h. Resultant nucleoside mixtures were lyophilized and reconstitute to final concentration of 1 ng/μl in RNase-free water, 0.1% formic acid for subsequent UHPLC-MS/MS analysis. A total of 3 instrument replicates were processed per sample. To quantify RNA modified nucleosides, calibration curves were prepared for 42 modified nucleosides including adenosine, cytidine, guanosine and uridine. [13C15N]-Guanosine was used as an internal standard. Several processing software scaffolds including MassLynx and Targetlynx (Waters, Milford, MA) were used for the post processing of UHPLC-MS/MS data. Python script / Production of calibration curves and the Originlab software suite (Northampton, MA) were used to quantify RNA modified nucleosides. Investigators were blinded to treatment throughout UHPLC-MS/MS procedures and analysis.

### Epididymosome isolation

Following the pelleting of motile cauda sperm (described above), epididymosomes were isolated from the supernatant by filtration and ultracentrifugation. First, the epididymosome-containing media was centrifuged at 10,000 × g for 30 min at 4°C before being passed through a 0.2 μm syringe filter. Finally, epididymosomes were pelleted in a table top ultracentrifuge at 120,000 × g for 2 h at 4°C, washed once with ice cold 1.5 ml PBS to remove excess protein aggregates, centrifuged again at 120,000 × g for 2 h at 4°C and snap frozen with liquid nitrogen.

### Epididymosome-sperm coincubation

The methods used for the coincubation were adapted from previously established methods (Reilly et al., 2016; Sharma et al., 2016). Briefly, for each paired sample, three 20-month-old adult male mice were sacrificed and each testis was dissected by removing the tunica and placing the seminiferous tubules in 3 ml HTF media at 37°C. The tissue was next finely minced and gently pipetted up and down to release spermatozoa and spermatogenic cells. After incubating further for 15 min at 37°C, sperm cells were run through a 100 μm cell strainer and centrifuged for 3 min at 200 × g to pellet somatic cells while leaving primarily immature sperm cells in suspension. This testicular spermatozoa-enriched preparation was pelleted at 1,000 × g and washed once in PBS. The sperm pellet was resuspended in 600 μl HTF (supplemented with 1 mM ZnCl_2_ and pH adjusted to 6.5) and half the sample was incubated for 3 h at 37°C with epididymosomes isolated from the whole epididymis of one mouse and the other half with an equal volume (50 μl) of epididymosome-depleted media from ultracentrifugation. Following coincubation, sperm were washed twice at 2,000 × g with PBS and immediately processed for RNA extraction.

### Electron microscopy

Exosome microscopy was performed with a JEOL JEM-1011 transmission electron microscope at Center for Biological Imaging at the University of Pittsburgh (Pittsburgh, PA) using negative staining procedures.

### Statistical analysis

Unpaired two-way student's *t*-tests were used to compare control and ethanol group means [body weight and all RT-qPCR experiments] and paired two-way *t*-tests were used for the sperm-epididymosome coincubation experiment. Two-way analysis of variance (ANOVA) was used to analyze distribution of tDR reads between control and ethanol groups for effects of ethanol or ethanol × tDR size. Bonferroni *post-hoc* tests were used to analyze specific group effects in the event of a significant ethanol × tDR size interaction. Analysis of nucleoside modifications was performed using multiple comparisons analysis (accounting for all 22 assessed nucleoside modifications in a single analysis) with false discovery rate adjustment using the two-stage step up method (*q* < 0.1) (Benjamini et al., 2006). Sequencing data was corrected for false discovery rate (*q* ≤ 0.1). Pearson's *r* was used to analyze all correlations between sperm and epididymosome tDRs for control and ethanol groups.

### Archiving sequencing data

Sequencing data was deposited to NCBI's Sequencing Read Archive with the accession: PRJNA414349.

## Ethics statement

This study was carried out in accordance with the recommendations of the National Institutes of Health Guidelines for the Care and Use of Laboratory Animals and Institutional Animal Care and Use Committee of the University of Pittsburgh. The protocol was approved by the Institutional Animal Care and Use Committee of the University of Pittsburgh.

## Author contributions

GR, IL, and GH designed the experiments. GR, AM, and QL performed the experiments. GR, QL, CW, IL, and GH analyzed the data. GR and GH wrote the manuscript.

### Conflict of interest statement

The authors declare that the research was conducted in the absence of any commercial or financial relationships that could be construed as a potential conflict of interest.

## Supplementary material

Supplementary Figure 1 Most tDR are 5′-derived tRNA halves. Graph showing the percentage of different tDR subtypes in chronic ethanol and control groups, classified by how each subtype is cleaved from the mature tRNA: 5′-derived tRNA halves (5′tRH), 5′-derived tRNA fragments (5′-tRF), 3′-derived tRNA halves (3′-tRH), 3′-derived tRNA fragments (3′-tRF), or undefined. Data presented as μ ± SEM.

Supplementary Figure 2 Effect of chronic ethanol on sperm piRNA. Volcano plot depicting fold change and log-transformed *p*-values for all piRNAs detected with small RNA sequencing. No piRNA species were significantly altered by chronic ethanol (*q* ≤ 0.1).

Supplementary Figure 3 Effect of chronic ethanol on blood ethanol concentration and body weights in sperm RNA modification analysis cohort. The average blood ethanol concentration (left panel) over the 5-week chronic ethanol exposure was 158.2 ± 6.8 mg/dl (μ ± SEM). Body weights (right panel) were not significantly altered by chronic ethanol exposure (*p* > 0.05). Data presented as μ ± SEM. *N* = 12/treatment.

Supplementary Figure 4 Epididymosomes transfer tDRs to immature testis sperm *in vitro*. Each testis sperm suspension was equally divided and half of the sample was incubated for 3 h with epididymosomes while the other half was incubated with epididymosome-depleted media to examine the effect of epididymosome *in vitro* coincubation on sperm tDRs **(A)** Gly-GCC and **(B)** Glu-CTC. Data presented as fold change from control values with lines indicating paired samples. ^*^*p* < 0.05.

Supplementary Figure 5 Relationship between sperm tDR and epididymosome tDR RT-qPCR expression. Scatterplots showing no correlation for levels of tDRs **(A)** Glu-CTC, **(B)** His-GTG, and **(C)** Ser-AGA expression between cauda sperm and cauda epididymosomes at 5 weeks and **(D)** a significant correlation at 2 weeks for tDR Glu-CTC in the ethanol group. Plotted lines for control and ethanol groups represent linear regression analysis. ^*^*p* < 0.05.

Supplementary Table 1 Differential expression analysis of sperm small noncoding RNA sequencing results (*n* = 9/group).

Supplementary Table 2 Predicted 3′-UTR targets of miRNAs altered in sperm by chronic ethanol exposure.

Supplementary Table 3 Gene ontology analysis of > 3 sperm miRNA that were increased by chronic ethanol exposure.

Supplementary Table 4 Predicted 5′-UTR, coding, and 3′-UTR targets of ethanol-responsive sperm tDR.

Supplementary Table 5 Gene ontology analysis for top predicted 5′-UTR targets of tDR Glu-CTC.

Supplementary Table 6 List of RNA modifications examined with UHPLC-MS/MS.

Supplementary Table 7 List of RT-qPCR oligos.
