# Supplementary figures and images for: Heavy Chronic Intermittent Ethanol Exposure Alters Small Noncoding RNAs in Mouse Sperm and Epididymosomes
Source: Front Genet. 2018 Feb 8;9:32. doi: 10.3389/fgene.2018.00032 (PMC5809758; doi:10.3389/fgene.2018.00032)

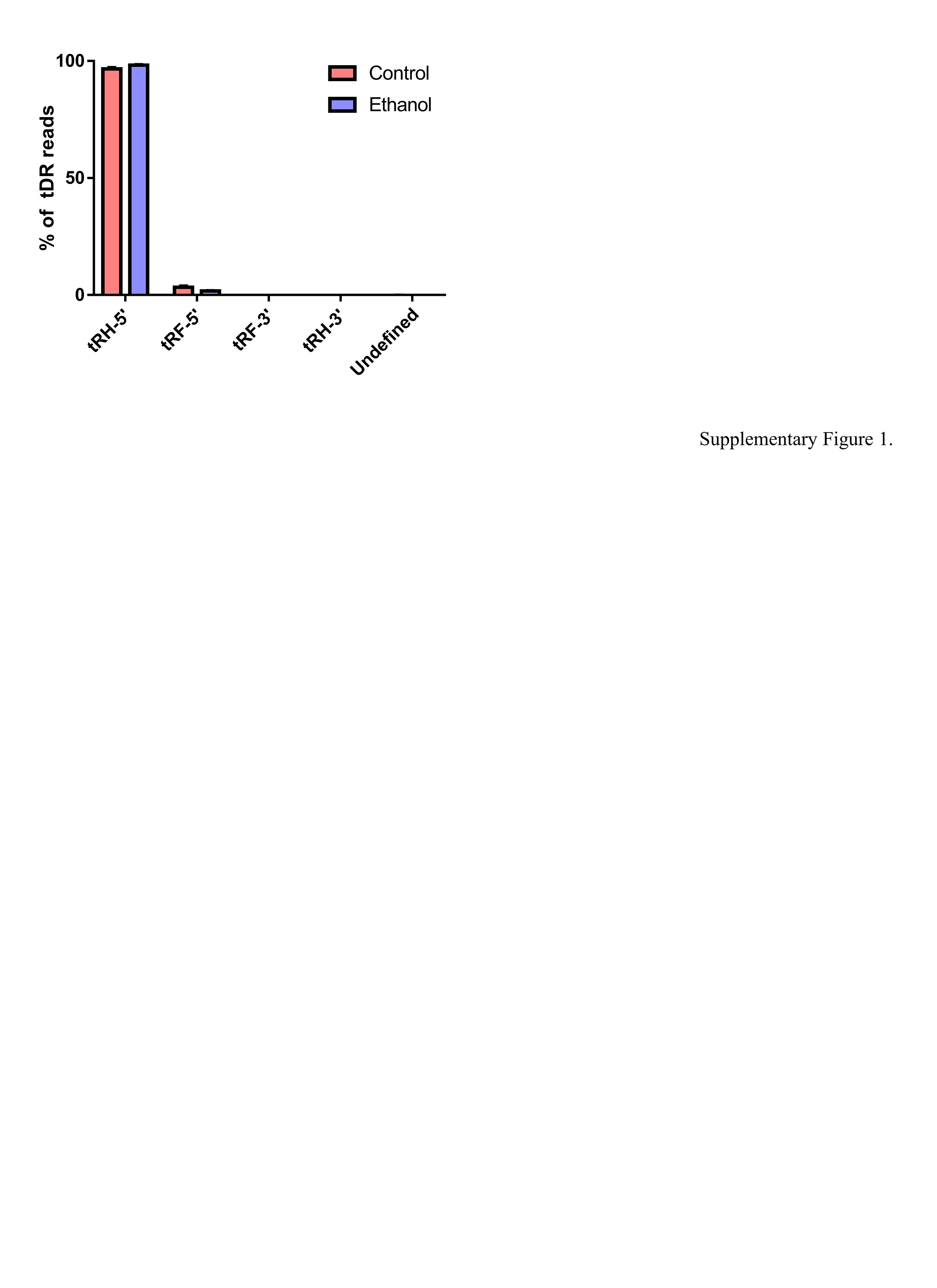

Supplement: Supplementary Figure 1 — Most tDR are 5′-derived tRNA halves. Graph showing the percentage of different tDR subtypes in chronic ethanol and control groups, classified by how each subtype is cleaved from the mature tRNA: 5′-derived tRNA halves (5′tRH), 5′-derived tRNA fragments (5′-tRF), 3′-derived tRNA halves (3′-tRH), 3′-derived tRNA fragments (3′-tRF), or undefined. Data presented as μ ± SEM. [file Image1.TIF]

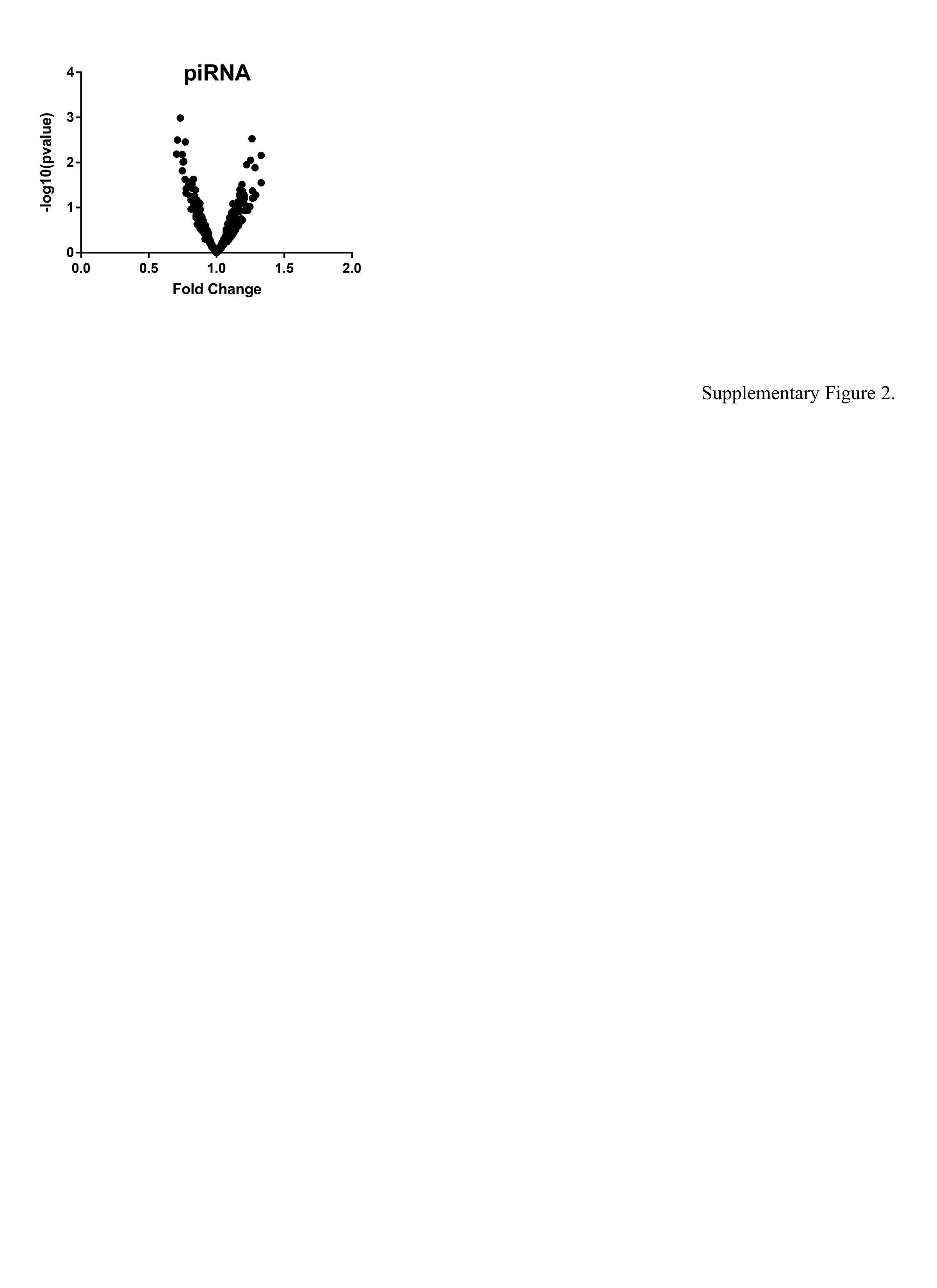

Supplement: Supplementary Figure 2 — Effect of chronic ethanol on sperm piRNA. Volcano plot depicting fold change and log-transformed p-values for all piRNAs detected with small RNA sequencing. No piRNA species were significantly altered by chronic ethanol (q ≤ 0.1). [file Image2.TIF]

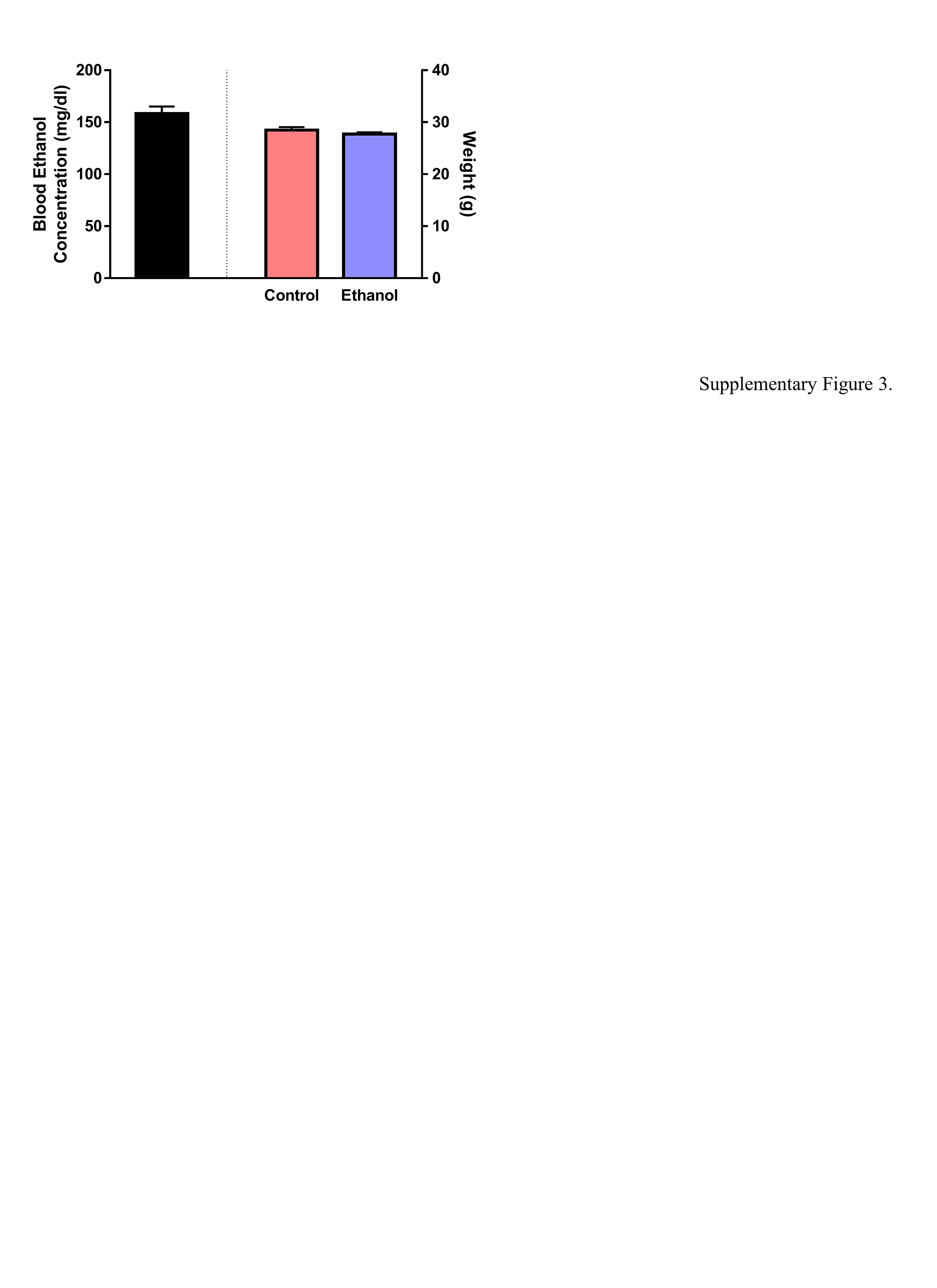

Supplement: Supplementary Figure 3 — Effect of chronic ethanol on blood ethanol concentration and body weights in sperm RNA modification analysis cohort. The average blood ethanol concentration (left panel) over the 5-week chronic ethanol exposure was 158.2 ± 6.8 mg/dl (μ ± SEM). Body weights (right panel) were not significantly altered by chronic ethanol exposure (p > 0.05). Data presented as μ ± SEM. N = 12/treatment. [file Image3.TIF]

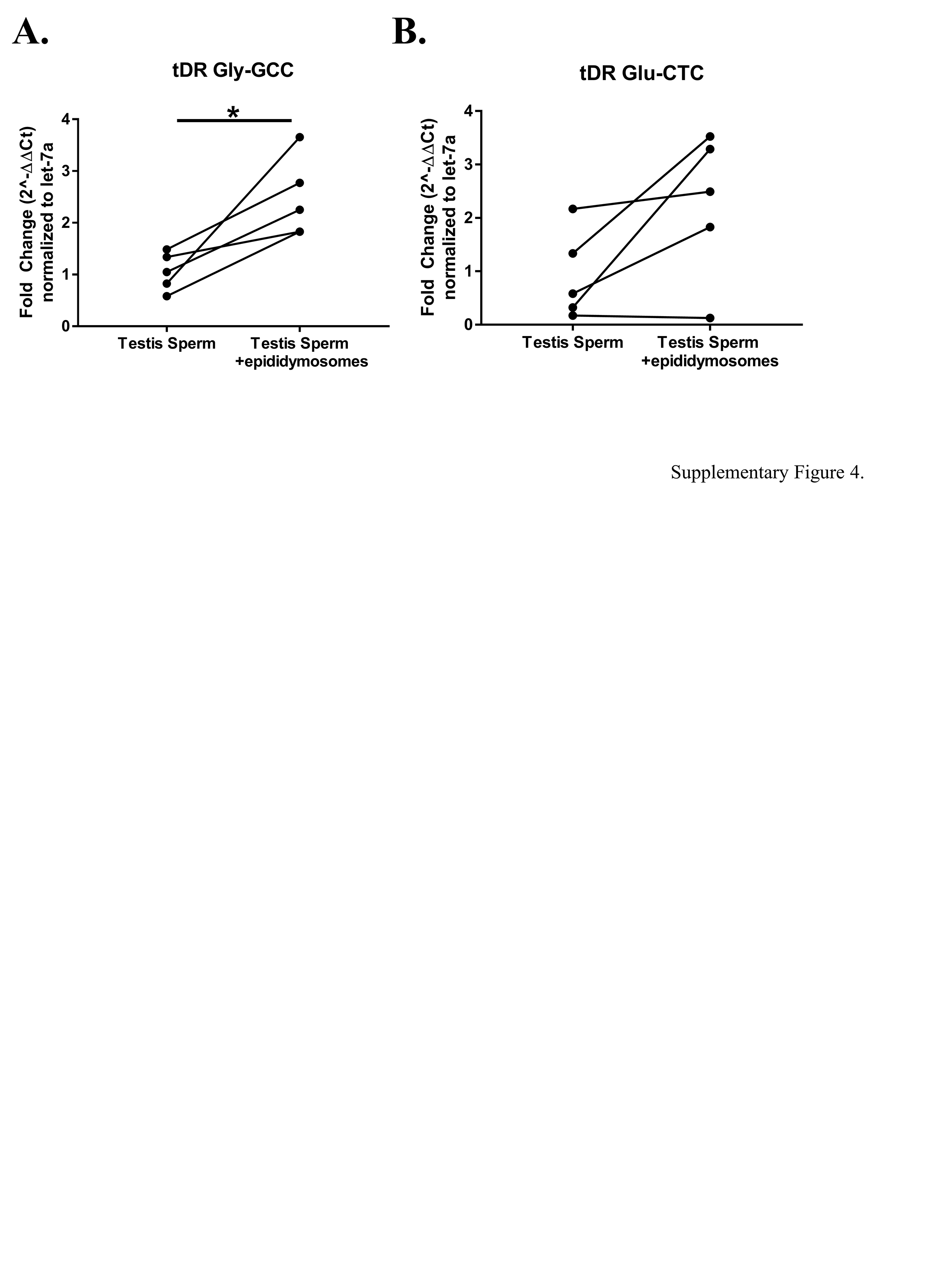

Supplement: Supplementary Figure 4 — Epididymosomes transfer tDRs to immature testis sperm in vitro. Each testis sperm suspension was equally divided and half of the sample was incubated for 3 h with epididymosomes while the other half was incubated with epididymosome-depleted media to examine the effect of epididymosome in vitro coincubation on sperm tDRs (A) Gly-GCC and (B) Glu-CTC. Data presented as fold change from control values with lines indicating paired samples. *p < 0.05. [file Image4.TIF]

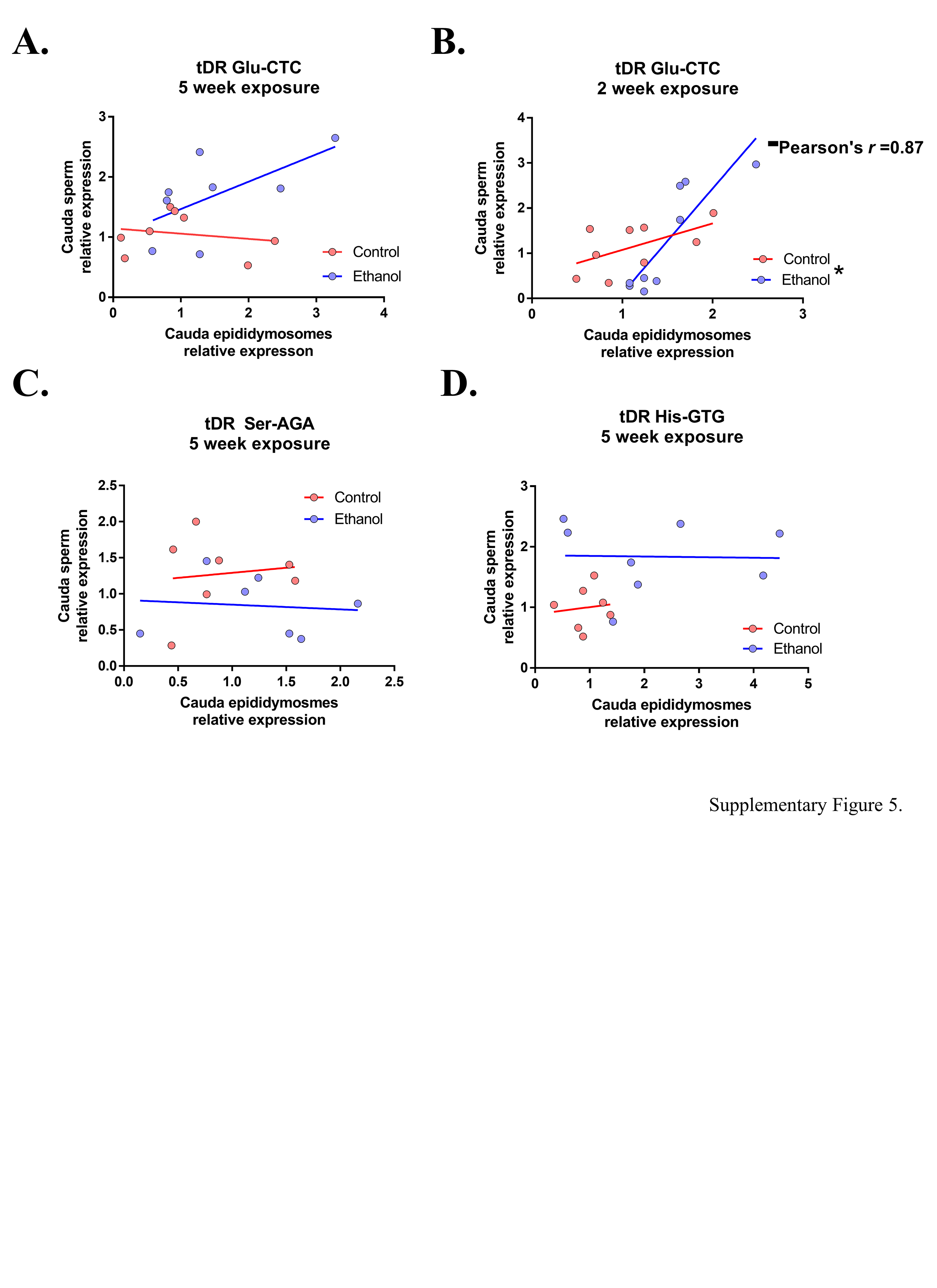

Supplement: Supplementary Figure 5 — Relationship between sperm tDR and epididymosome tDR RT-qPCR expression. Scatterplots showing no correlation for levels of tDRs (A) Glu-CTC, (B) His-GTG, and (C) Ser-AGA expression between cauda sperm and cauda epididymosomes at 5 weeks and (D) a significant correlation at 2 weeks for tDR Glu-CTC in the ethanol group. Plotted lines for control and ethanol groups represent linear regression analysis. *p < 0.05. [file Image5.TIF]
